# Concave-convex Reaming of Intercalary Allograft: 1-year Clinical Outcomes

**DOI:** 10.5435/JAAOSGlobal-D-20-00023

**Published:** 2021-04-20

**Authors:** Nathan Bastien, Sean Kelly, Dustin Lybeck

**Affiliations:** From the Orthopaedic Surgery, Brooke Army Medical Center, San Antonio, TX.

## Abstract

Complication rates associated with intercalary allograft reconstruction may be reduced by maximizing tenants of allograft reconstruction. Intercalary allograft reconstruction using a hemispherical reaming technique for graft-host interface may increase surface contact areas, provide intimate contact between surfaces, and equally distribute pressure subsequently decreasing risk of nonunion. The purpose of these case reports was to present short-term results for limb salvage using this novel technique for two young, active duty military members who returned to full-impact activity.

Reconstruction of lower extremity diaphyseal tumor resections includes intercalary allograft, prosthetic reconstruction, and amputation. Allograft reconstruction has the advantage of maintaining bone stock in younger patients and may avoid the late complication of aseptic hardware loosening seen in prosthetic reconstructions. However, lack of incorporation of the intercalary bone graft can present several unique problems. Nearly half of patients experience a delayed union, defined as no radiographic union by 1 year after surgery, and graft fracture has been reported to occur in up to 30% of patients.^1^ In addition, nonunion, defined as the cessation of both periosteal and endosteal healing responses without bridging bone,^2^ approaches 40% in allograft reconstruction.^3^ Tibial intercalary allografts (compared with femoral) and allografts under 10 cm have been associated with decreased nonunion rates, whereas isolated nail fixation has been associated with increased nonunion risk.^3^ Nonunion has been reported to result in up to a 40% reoperation rate resulting in notable patient morbidity.^3^ Union is achieved on average at 12 months with a decreased time for metaphyseal-metaphyseal versus diaphyseal-diaphyseal junctions at 9.1 and 16.3 months on average, respectively.^4,5^ Tenets of allograft fixation for promotion of bone healing and subsequent reduction in nonunion include maximizing contact surface area, stable fixation, and equal distribution of compression across the graft and host interface.

Several techniques have been developed to promote union including transverse cut, step-cut, sigmoid cut, and concave-convex reaming; however, there is a paucity of literature comparing union rates and time to union of concave-convex reaming versus former methods.^6,7^ When compared with transverse osteotomies, the sigmoid and step-cut provide 74% and 44% greater contact surface area, respectively.^5^ The concave-convex reamed interface provides more than doubled the surface contact area, when compared with a transverse cut.^4^ Concave-convex osteotomy was also more effective in comparison with transverse osteotomies in evenly distributing pressure across the graft-host interface, which may prevent stress concentration and subsequent fracture.^4,5^ Clinical results in the literature are limited to one technique article reporting the successful implementation of the reaming technique in two allograft patients.^8^

We hypothesized that concave-convex reamed intercalary allografts provide a biomechanical advantage over traditional osteotomies and may provide a durable enough construct to return to full-impact activity. We present the clinical results of concave-convex intercalary allograft reconstruction in two active duty military patients, with return to full duty, after diaphyseal tibia resection for primary bone sarcoma with intermediate-term results.

## Cases

### Case 1

**Figure 1:**
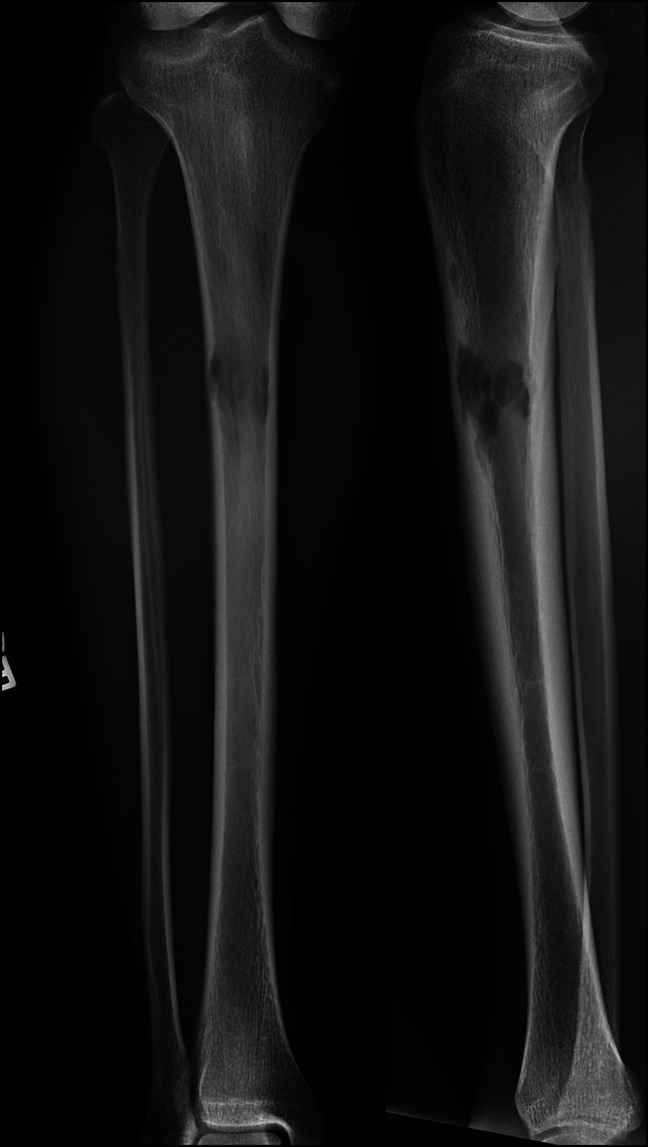
Radiograph of 27-year-old man demonstrating poorly defined lytic lesion in diaphysis of tibia with endosteal scalloping (case 1: AP and lateral radiographs of diaphyseal tibia lesion).

**Figure 2:**
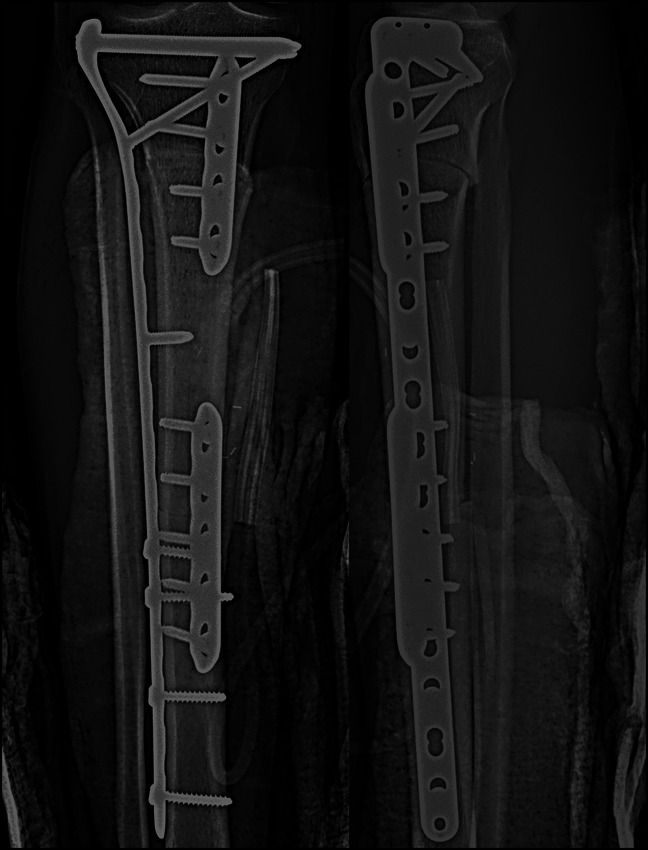
Radiograph of 27-year-old man demonstrating hemispherical reaming at metadiaphysis and mid-diaphysis graft-host interface (case 1: AP and lateral radiographs after intercalary allograft reconstruction on postoperative day zero). Interfaces are compressed with low-contact dynamic compression plates with bridge plate expansion of entire construct.

A 27-year-old active-duty male infantry soldier presented with right leg pain after a physical fitness test and a presumed diagnosis of a tibial stress injury. Initial radiographs demonstrated a lucent lesion of the mid-diaphyseal tibia with biopsy and staging consistent with an isolated high-grade osteosarcoma of the tibia (Figure 1). He then underwent two cycles of neoadjuvant chemotherapy per AOST0331 (doxorubicin/cisplatin/high-dose methotrexate), wide resection (12 cm), and a concave-convex intercalary allograft reconstruction (Figure 2) with hemisoleus rotation flap followed by an additional four cycles of chemotherapy. Negative margins were achieved with 80% tumor necrosis.

The tumor was resected using standard transverse cuts based on preoperative measurements, and reconstruction was undertaken after intraoperative pathology confirmed disease-free margins. The proximal and distal aspects of the host tibia were then shaped with 53 mm and 44 mm acetabular reamers, respectively. The fresh-frozen, size-matched allograft (Musculoskeletal Transplant Foundation, Edison, New Jersey) was prepared on the back table and held with bone reduction forceps during preparation. Similar to the technique described by Wilkes et al,^6^ the allograft was first cut transversely with 1 cm of additional length at both the proximal and distal ends to allow reaming. Complementary hemispherical reaming of the allograft tissue was done using a reverse reamer from the Equinoxe shoulder resurfacing system (Exactech); reamer sizes are available in 3 mm increments from 38 to 53 mm. Normal saline irrigation was used for lubrication and to aid in temperature reduction during reaming. Allograft fit was grossly assessed frequently in vivo. The graft was then secured with hybrid locking-compression plating.

Compression of the plate and a graduated progression of weight bearing was believed to allow for a stress healing response without overloading the initial avascular, distal ends of the allograft while allowing for soft-tissue healing. Subsequently, our postoperative protocol required monthly radiographs and limited weight bearing until union as described by Ortiz-Cruz et al^9^ with several changes. The patient remained non–weight bearing for 6 weeks postoperatively followed by an additional 6 weeks of toe-touch weight bearing. After 12 weeks, the patient was advanced to 50% weight bearing and began therapy with the use of a weight-limiting, low-impact treadmill (AlterG). Patients were progressed to full weight bearing once either the patient was pain free, and radiographs demonstrated osseous bridging or 16 weeks had elapsed since surgery. Full-impact activity was not permitted until there was osseous bridging at both the graft interfaces.

Osseous bridging was noted at the proximal tibial metaphyseal interface 12 weeks postoperatively and the distal diaphyseal interface 28 weeks postoperatively. A computed tomography (CT)-guided biopsy of the distal host bone tibia was obtained 32 weeks postoperatively to evaluate for a possible recurrence noted on radiographs. The histology demonstrated benign, fibrous tissue. The CT scan allowed for additional characterization of the host allograft interface confirming healing of the proximal junction with near circumferential union of the distal junction except for the anteromedial 20%.

**Figure 3:**
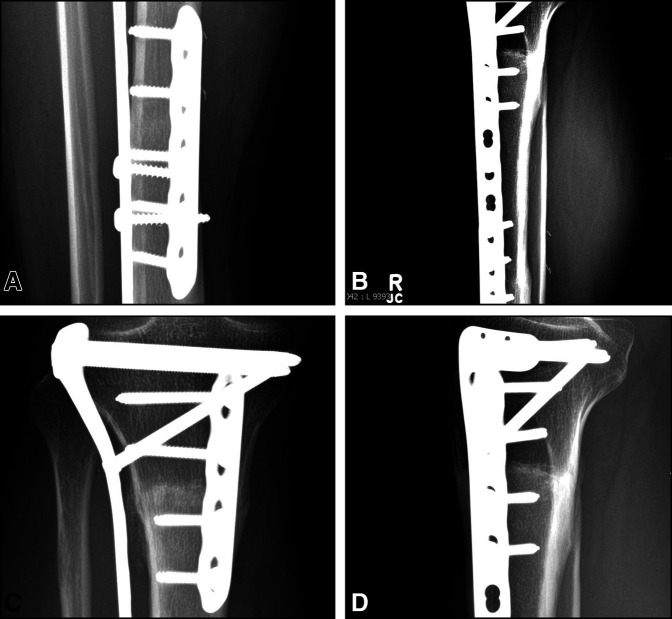
Radiograph showing AP and lateral views. **A**, Case 1: AP radiograph of distal tibia demonstrating host-allograft junction union at the 33-month follow-up. **B**, Case 1: lateral radiograph of distal tibia diaphyseal host-allograft junction demonstrating union at the 33-month follow-up. **C**, Case 1: AP radiograph of proximal tibia demonstrating host-allograft junction union at the 33-month follow-up. **D**, Case 1: lateral radiograph of proximal tibia demonstrating host-allograft junction union at the 33-month follow-up.

The patient was advanced to full weight bearing at 16 weeks and was running with the assistance of an Intrepid Dynamic Exoskeletal Orthosis (US Army) at 36 weeks postoperatively. At the last follow-up (33 months), the patient had returned to full active duty including marching with a full combat-load (70 pounds) without pain or evidence of construct failure (Figure 3).

### Case 2

**Figure 4:**
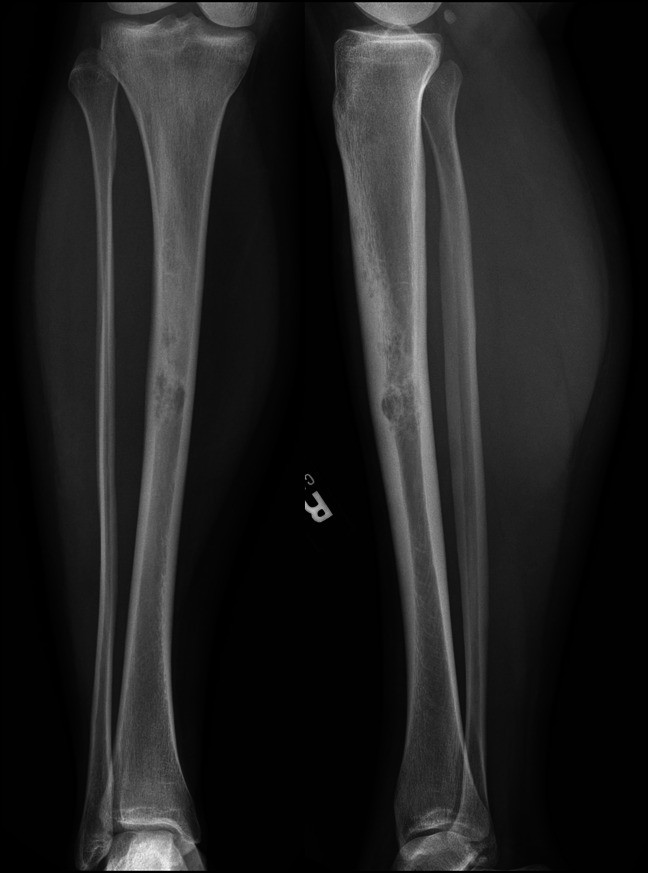
Radiograph of 28-year-old woman demonstrating poorly defined, mixed, cortically based lesion with endosteal scalloping without apparent cortical destruction (case 2: AP and lateral radiographs of diaphyseal tibia lesion).

A 28 year-old active duty women presented with bilateral anterior leg pain during basic training and was found to have a 10-cm cortically based lucent lesion (Figure 4). Biopsy proved the bone lesion to be an adamantinoma, and staging studies were negative for metastatic disease.

**Figure 5:**
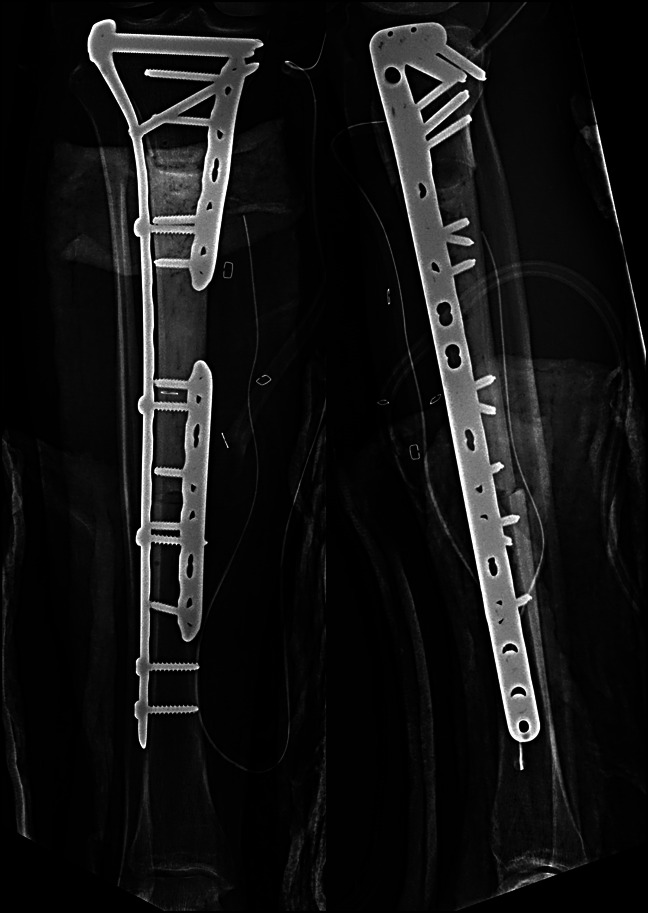
Radiograph of a 28-year-old woman demonstrating hemispherical reaming at proximal tibia metadiaphysis and mid-diaphysis graft-host interface with low-contact dynamic compression plate fixation (case 2: AP and lateral radiographs after intercalary allograft reconstruction on postoperative day zero).

Wide resection and intercalary allograft reconstruction were done as described in case 1 with a 14-cm tibial resection (Figure 5). During preparation of the proximal allograft, the convex reamer chipped and thinned the allograft, resulting in decreased cortical thickness. Polymethylmethacrylate bone cement was placed into the intramedullary canal of the proximal allograft to reinforce the thinned cortex and provide additional rigidity; the host-allograft junction was free of cement to avoid interference with graft-host cortical apposition. A medial gastrocnemius rotational flap and split-thickness skin grafting were done for soft-tissue coverage. On postoperative day 2, the patient developed a compartment syndrome and underwent anterior and lateral compartment releases further complicated by a superficial skin infection on day 14 that resolved with oral antibiotics.

**Figure 6:**
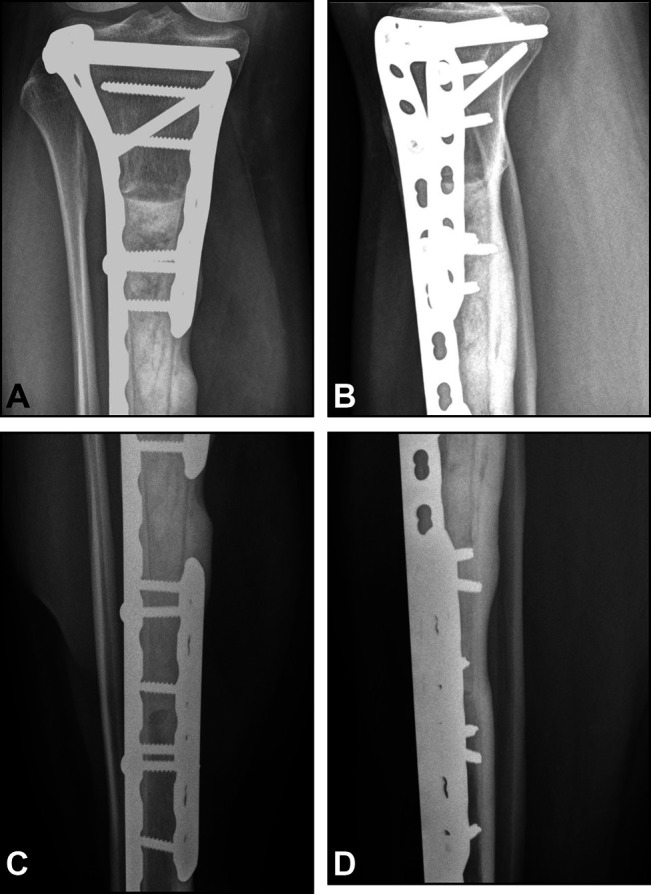
Radiograph showing AP and lateral views. **A**, Case 2: AP radiograph of proximal tibia and previous allograft fracture 34 months postoperatively demonstrating union of host-allograft junction and allograft fracture. **B**, Case 2: lateral radiograph of proximal tibia and previous allograft fracture 34 months postoperatively demonstrating union of host-allograft junction and allograft fracture. **C**, Case 2: AP radiograph of distal tibia and previous allograft fracture 34 months postoperatively demonstrating union of host-allograft junction and allograft fracture. **D**, Case 2: lateral radiograph of distal tibia and previous allograft fracture 34 months postoperatively demonstrating union of host-allograft junction and allograft fracture.

The patient self-progressed to 50% weight bearing at 6 weeks, 6 weeks before our protocol advancement that occurs with the addition of weight-limited treadmill running. Full weight bearing was delayed by the primary investigator for an additional 4 weeks to offset the early progression. Subsequently, the patient was advanced to full, low-impact weight bearing 20 weeks postoperatively. Osseous bridging was identified at the proximal junction at 20 weeks and the distal diaphyseal-allograft junction at 28 weeks postoperatively, and she was then advanced to full-impact, per protocol. However, at 36 weeks postoperatively, the patient developed a painless periosteal reaction at the mid-posterior cortex of the allograft and reported she had self-progressed to full-impact 20 weeks postoperatively. A CT scan was obtained identifying the appearance of a stress fracture with associated periosteal reaction. The patient was then kept at 50% weight bearing and prescribed low-intensity ultrasonography bone stimulation for 12 weeks before radiographs and CT imaging demonstrated fracture healing. At 12 months postoperative, she was able to run one mile without pain or assistive device. At 34 months, the patient has no activity limitations and no further graft complication on radiographs (Figure 6).

## Discussion

In young, active patients, the ideal reconstruction after diaphyseal resection has yet to be determined. As both our patients were active duty soldiers intending to return to full duty, both prosthetic reconstruction and amputation were not feasible options. Concave-convex reaming was used in our patients in an effort to maximize graft healing.

A hybrid plate construct was chosen to compress the graft-host interface site, and cement was used in one patient to reinforce an allograft with thin cortices. This was described by Gerrand et al^10^ and Gupta et al^11^; both reported intramedullary cement augmentation may improve the mechanical properties of intercalary allografts and decrease risk of fracture without impeding union. The addition of intramedullary nail fixation may have provided additional load sharing, but was felt to limit the screw trajectory, and thus, the compression we could achieve. The use of vascularized fibular graft may have prevented the occurrence of graft fracture and shortened time to union;^12^ however, donor site morbidity was a major concern.

We presume that our patient's allograft fractured despite cement augmentation because she prematurely advanced to full-impact activity. However, the fracture occurred at the allograft-cement interface where healing may have been delayed and predisposed her to injury; a clinicopathological study that included five specimens composed of cement-allograft composite demonstrated no histologic evidence of revascularized allograft adjacent to cement or ingrowth of tissue between the cement and adjacent allograft.^5^ In addition, retrieval of allograft specimens has demonstrated less than a few millimeters of intramedullary reparative tissue formation within the first year of reconstruction, followed by typically no more than 2 cm of new bone laid over necrotic allograft endosteum.^5^ This suggests that intramedullary cement augmentation may be effective in establishing a mechanically sound construct when deficient allograft exists but may interfere with effective graft incorporation required to withstand forces experienced with impact activity.

We observed decreased time to union at the meta-diaphyseal versus diaphyseal-diaphyseal host-allograft interfaces as expected by previous studies^5,9,13,14^; the patients demonstrated osseous bridging of the proximal meta-diaphyseal interface at 12 and 20 weeks and diaphyseal-diaphyseal junction at 28 weeks. However, the second patient sustained an allograft fracture after premature resumption of full-impact activity 20 weeks postoperative. This supports the need for prolonged activity restrictions despite presumed osseous union. Both patients maintained their grafts at an average of 33.5 months despite returning to full-impact activity. Overall 5-year construct survival for intercalary allografts has been reported up to 79% to 84% with no difference in patients receiving chemotherapy^13^; however, patients undergoing chemotherapy, radiation of the tumor bed, or both were noted to have increased rate of nonunion, fracture, and infection^9^ indicating a potential role for vascularized grafts in this patient subset. Infection rates for intercalary allografts are high (5% to 50%)^3,11-13^ resulting in amputation rates of up to 20%^15^ which we would likely encounter more frequently with a larger patient cohort and was not related to our surgical technique.

Functional outcomes in patients after diaphyseal tibial resections are promising after intercalary allograft reconstruction^8^ when compared with amputation.^16^ However, in a subset of young patients, amputation may be a more attractive option to allow full weight bearing and impact activities. Attempts at activity after allograft reconstruction should only be permitted after a thorough risk-benefit discussion. It should be noted that our patients would not have been able to continue in their current career with either amputation or activity restriction necessitating a trial of full activity including running, jumping, and carrying loads.

Limitations in this study include patient volume, lack of long-term follow-up, or a comparison group. In addition, patient selection may have had a notable impact on their outcome because these both were physically fit, highly functional individuals with access to state-of-the art rehabilitation. The contribution from the surgical procedure is hard to quantify because our time to union was not unexpectedly different from historical controls and we had no comparison group; however, at the final follow-up, we demonstrated that two adult patients with intercalary tibial allografts were able to maintain a previously unreported level of function with this surgical technique. Further research is required before widespread adoption of this technique and would include larger prospective, comparative studies including multiple conventional graft preparation techniques. Reconstruction options must continue to address this gap in the treatment armamentarium to provide patients with a durable yet functional limb that supports the aspirations of the young and active but will endure into old age.

## Conclusion

To the best of the author's knowledge, this is the first to report of adult sarcoma patients returning to running and impact activity after intercalary allograft reconstruction. The contribution of concave-convex reamed junctions to these patients' outcomes cannot be determined from this limited case series, but the durability demonstrated in these two constructions should encourage future prospective, controlled studies to determine the ideal construct for highly active patients undergoing bone sarcoma resection.
